# Assessing Sunscreen Protection Using UV Photography: Descriptive Study

**DOI:** 10.2196/24653

**Published:** 2021-05-26

**Authors:** Caitlin Horsham, Helen Ford, Jeremy Herbert, Alexander Wall, Sebastian Walpole, Elke Hacker

**Affiliations:** 1 School of Public Health and Social Work Queensland University of Technology Brisbane Australia; 2 Designworks Group Pty Ltd Brisbane Australia; 3 Genetics & Population Health Division QIMR Berghofer Brisbane Australia

**Keywords:** skin neoplasms, melanoma, health promotion, public health, preventive medicine, sunburn, sunscreening agents, UV photography, mobile phone

## Abstract

**Background:**

Photography using a UV transmitting filter allows UV light to pass and can be used to illuminate UV blocking lotions such as sunscreens.

**Objective:**

The aim of this study is to compare currently available UV photography cameras and assess whether these devices can be used as visualization tools for adequate coverage of sun protection lotions.

**Methods:**

This study was conducted in 3 parts: in phase 1, 3 different UV cameras were tested; in phase 2, we explored whether UV photography could work on a range of sun protection products; and in phase 3, a UV webcam was developed and was field-tested in a beach setting. In phase 1, volunteers were recruited, and researchers applied 3 sun protection products (ranging from sun protection factor [SPF] 15 to 50+) to the participants’ faces and arms. UV photography was performed using 3 UV cameras, and the subsequent images were compared. In phase 2, volunteers were recruited and asked to apply their own SPF products to their faces in their usual manner. UV photographs were collected in the morning and afternoon to assess whether the coverage remained over time. Qualitative interviews were conducted to assess the participants’ level of satisfaction with the UV image. In phase 3, a small portable UV webcam was designed using a plug-and-play approach to enable the viewing of UV images on a larger screen. The developed webcam was deployed at a public beach setting for use by the public for 7 days.

**Results:**

The 3 UV camera systems tested during phase 1 identified the application of a range of sun protection lotions of SPF 15 to 50+. The sensitivity of the UV camera devices was shown to be adequate, with SPF-containing products applied at concentrations of 2 and 1 mg/cm^2^ clearly visible and SPF-containing products applied at a concentration of 0.4 mg/cm^2^ having lower levels of coverage. Participants in phase 2 reported high satisfaction with the UV photography images, with 83% (29/35) of participants likely to use UV photography in the future. During phase 2, it was noted that many participants used tinted SPF-containing cosmetics, and several tinted products were further tested. However, it was observed that UV photography could not identify the areas missed for all tinted products. During phase 3, the electrical components of the UV webcam remained operational, and the camera was used 233 times by the public during field-testing.

**Conclusions:**

In this study, we found that UV photography could identify the areas missed by sun protection lotions with chemical filters, and participants were engaged with personalized feedback.

**Trial Registration:**

Australian New Zealand Clinical Trials Registry (ANZCTR) ACTRN12619000975190; http://www.anzctr.org.au/Trial/Registration/TrialReview.aspx?id=377089 ; Australian New Zealand Clinical Trials Registry (ANZCTR) ACTRN12619000145101; https://www.anzctr.org.au/Trial/Registration/TrialReview.aspx?id=376672.

## Introduction

### Background

Reflected UV photography provides a unique method of assessing sunscreen application. A camera using a UV transmitting filter allows UV radiation to pass but absorbs or blocks visible and infrared light. The subject is illuminated by either UV emitting lamps or sunlight, and a photo is taken, which then highlights the areas where sunscreens have been applied. Sunscreen application followed by UV photography is a potential method to objectively measure the visibility of sunscreen on the skin [1]. Pratt et al [2] have shown that UV photography can detect commonly missed areas during sunscreen application on the face, with participants missing the eyelids and the medial canthal area around the eyes. Molecular analysis of normal eyelids has also shown that over a quarter of cells carry mutations that exhibit characteristic signatures of UV light exposure [3]. The eyebrow and eyelid have also been reported as high-risk anatomical sites for locally destructive basal cell carcinoma skin cancers [4].

Skin cancer is estimated to account for more cases diagnosed than all other cancers combined in Australia, costing over Aus $800 million (US $622 million) to treat each year [5-7]. Sunlight or UV radiation is the main risk factor for skin cancers, and sunburn remains highly prevalent in the northern Australian state of Queensland, with 49% of adults and 45% of children sunburnt in the previous 12 months [8]. Of the children who were sunburnt in the past 12 months, 69% were most recently sunburnt during a water-based activity [8]. These findings are concerning and highlight the importance of adequate sunscreen coverage and reapplication when participating in water-based activities. Regular sunscreen application has been shown to reduce the incidence of squamous cell carcinoma and melanoma [9,10] and block the harmful molecular effects of UV radiation on skin cells in vivo [11].

Barriers reported to sunscreen application include concerns over sunscreen esthetics and tactile properties, including a sticky or greasy texture, feeling hot or sweaty, perception that sunscreens cause acne or skin irritation, and dislike of sunscreen appearance [12]. Many cosmetic products are secondary sunscreens with a sun protection factor (SPF) that offers convenience and improved texture and appearance. In Australia, the industry-accepted SPF standard tests primary sunscreens as well as secondary sunscreen products, which are applied at a thickness of 2 mg/cm^2^ and rated for SPF from 0 to 50+. For SPF products to be effective, adequate quantities of the product need to be applied with an appropriate frequency of reapplication. There are 2 types of sun protection formulations: (1) physical filters such as titanium dioxide or zinc oxide, which act by scattering sunlight from the skin surface, or (2) chemical filters that transform the energy from the sun into molecular conformational changes [13]. Physical filters cannot be detected with UV photography, and only lotions with chemical filters can be visualized. In addition, some cosmetic products are tinted and contain both chemical and physical filters in their formulations. The physical filters within these products may limit their ability to be visualized. Tinted SPF cosmetics contain a temporary color or pigmentation and can include products such as foundations, lipsticks, and eye shadows.

### Objectives

The purpose of this study was to compare UV photography cameras and assess whether these devices can be used as visualization tools for adequate coverage of a range of SPF lotions commonly applied to the face, including sunscreens, moisturizers, and cosmetics.

## Methods

This study was conducted in 3 parts: phase 1 was laboratory testing, which involved testing different UV cameras and SPF product coverage; phase 2 was determining whether UV photography could visualize a range of sun protection products self-applied by individuals; and phase 3 was developing a UV webcam and field-testing the device in a public beach setting. 

### Phase 1: Laboratory Testing of UV Cameras

Commercially available UV cameras were purchased using the purchasing protocol, which involved searching the internet using the terms “UV camera,” “sunscreen detector,” and “sunscreen camera.” A total of 3 devices were identified and purchased for delivery to Australia. The cameras of 2 devices, the Sunscreenr (Vocelight LLC) and Nurugo SPF (Nurugo), attach to Android smartphones and are used in combination with an app. The third device used a digital single-lens reflex (DSLR) camera (Model D5300, Nikon) fitted with a Baader Venus filter (Model Baader Planetarium U-Filter 2“, Ultraviolet, ZWL 350 nm).

Participants were eligible to participate if they were aged 18 years or above and were available to attend the university campus. Participants were excluded if they had allergies or were sensitive to sunscreen. The sample size calculation for phase 1 was based on the recommendations from the industry-accepted Sunscreen Standard (AS/NZS 2604:2012), which sets a minimum sample size of 10 participants to assess each sun protection product. Participants were recruited through university email and social media posts. Participants completed a demographic survey and removed any skin care or makeup products from their face and arms using isopropanol wipes and paper towels. Images of the treatment sites (face and forearms where SPF lotions were applied) were captured using the DSLR UV camera and normal photography before any lotions were applied. This was to ensure that there were no SPF lotions on the skin before treatment. The treatment areas were marked by the researchers using plastic cutout rectangles (4×2.5 cm) on the participants’ face and both forearms. Each SPF lotion was randomly assigned to a treatment site and applied at concentrations of 2, 1, and 0.4 mg/cm^2^ (Figure S1 in Multimedia Appendix 1). The SPF lotions used included (1) sunscreen SPF 50+ (Cancer Council Ultra; active ingredients: homosalate 100 mg/g, octyl salicylate 50 mg/g, butyl methoxydibenzoylmethane 30 mg/g, and octocrylene 80 mg/g), (2) moisturizer secondary sunscreen SPF 50+ (SunSense Moisturizer; active ingredients: bemotrizinol 2%, methylene bis-benzotriazolyl tetramethylbutylphenol 2.5%, and octyl salicylate 5.0%), and (3) moisturizer secondary sunscreen SPF 15+ (Neutrogena Moisturizer; active ingredients: butyl methoxydibenzoylmethane, ethylhexyl methoxycinnamate, ethylhexyl salicylate, and phenylbenzimidazole sulfonic acid). Only SPF lotion products with chemical sunscreen filters were used, as zinc oxide and titanium dioxide cannot be visualized with UV photography. Images were captured immediately after SPF product application (baseline timepoint) and 20 minutes post application (follow-up timepoint) using the 3 UV cameras purchased. UV images of the participants’ faces (front, left side, and right side) as well as both forearms were taken at each timepoint.

Image analysis was performed using Image J (National Institutes of Health) [14], and the scale-to-pixel measurement was assigned using the treatment area (4×2.5 cm), with the rectangle tool used to define the region of interest. Image thresholds were set, and the percentage of area with dark pixels (SPF lotion present) were compared with the percentage of area with light pixels (no SPF lotion present) to calculate the percentage of coverage.

### Phase 2: Testing UV Photography Using a Range of Sun Protection Products

#### Part 2a: Observational Study

An observational study was conducted to assess the application of sun protection products of indoor workers. To be eligible, participants had to be aged 18 years or above, a current indoor worker, routinely use products with SPF on their face, and available to visit the researchers to attend both morning and afternoon photo sessions on the same day. Participants were recruited through email, social media, and the Queensland University of Technology workplace health and safety programs. Participants provided consent, completed a baseline questionnaire, and were asked to apply their own SPF products to their face in their usual manner before attending the study visit. To assess if the coverage remained over time, participants were imaged in the morning and then again in the afternoon, with a gap of at least 4 hours between timepoints. A total of 3 UV images of the participants’ faces (front, left side, and right side) were taken at each timepoint. Images were captured indoors using a white background and standard lighting, with participants sitting on a stool at a set distance from the camera, and an artificial UV light source (Nurugo) was used for UV illumination. In the afternoon, participants were shown their UV images, and an in-person interview was conducted. During the interview, participants were asked about their level of satisfaction with their UV photography images. The interview questions are listed in Table S1 in Multimedia Appendix 1.

To assess the difference in coverage between the morning and afternoon photo sessions, an automated image analysis method was developed to objectively detect, segment, and quantify the areas of the face within the UV images that were not adequately covered by SPF lotions. Volocity 3D image analysis software (PerkinElmer) was used. A scale-to-pixel measurement was assigned to each image using the ID sticker (19×24 mm), the region of interest tool was imposed, and the find object tool was used to find the percentage of area with dark pixels (SPF lotion present) and compared this with the percentage of area with light pixels (no SPF lotion present). The segmented areas included the nose, cheeks, forehead, and medial canthal area, which were then scored as “yes, adequately protected” or “no, not adequately protected.”

#### Part 2b: Testing Tinted SPF Lotions

Many SPF lotions used by participants in the observational study were tinted products that combined a colored base with SPF protection. Commonly used tinted sun protection products by participants in the observational study were purchased by the researchers for further laboratory testing in 1 volunteer. The 5 products used included (1) Fit Me SPF 18 liquid foundation (*Maybelline*; *active ingredients: octinoxate 7%*), (2) Lasting Radiance SPF 28 liquid foundation (*Rimmel*; *active ingredients: octinoxate*), (3) SkinActive beauty balm (BB) cream SPF 15 (*Garnier*; *active ingredients: octinoxate*), (4) BB cream SPF 15 (*Olay*; *active ingredients: octisalate* and *avobenzone*), and (5) SkinActive BB cream SPF 50+ (*La Roche-Posay*; *active ingredients: homosalate 6.0% w/w, octyl salicylate 5.0% w/w, butyl methoxydibenzoylmethane 5.0% w/w, octocrylene 5.0% w/w, ethylhexyl triazone 4.0% w/w, bemotrizinol 3.0% w/w, drometrizole trisiloxane 3.0% w/w, ecamsule 0.99% w/w,* and *titanium dioxide 0.83% w/w*). The volunteer provided informed consent, completed a demographic survey, and was asked to visit the researchers at the university. At the study visit, the volunteer was asked to remove any skin care or makeup products using isopropanol wipes and then rinse the area using running water and a paper towel. A UV image and normal photography image were taken from the treatment site before application to ensure that no lotions remained on the skin. The participants and research staff were blinded to the brand and SPF strength of the lotion. Lotions were applied to participants’ forearms to compare the 5 products at 2, 1, 0.4, and 0.2 mg/cm^2^ concentrations each to a 4×2.5 cm area of skin. Tinted sun protection products 4 and 5 were further evaluated at lower concentrations on the face, with applications of 1, 0.6_,_ 0.4, and 0.2 mg/cm^2^. Data collection included images captured immediately after application using both the DSLR UV camera and a normal camera (Nikon).

Phases 1 and 2 of the study were approved by the Human Research Ethics Committee of the Queensland University of Technology (number 1800001263) and prospectively registered with the Australian and New Zealand Clinical Trials Register (ACTRN12619000975190; ACTRN12619000145101). The sample size calculation for the phase 2 observational study was based on the recommendations from Lancaster et al [15] of 30 participants, which is widely used in feasibility testing studies.

### Phase 3: Development, Safety, and Field-Testing of a UV Webcam

#### Development

A UV webcam on a large screen was developed for use at public events, which could be used by the public with contactless operation. The UV webcam was developed using a UV transmitting filter (Edmund Optics) and an M12 lens (ArduCam) connected to a printed circuit board for processing electronics and housed within a plastic molding with a 365-nm UV light-emitting diode light source. A high-definition multimedia interface output cable was used to display the image, and a commercially available pressure sensor mat (Radio Parts Pty Ltd) was connected via a custom data acquisition system to report the pressure-sensitive switch information in real time over a USB connection. The pressure sensor mat allowed use data to be collected and stored data locally on a microSD card. The UV webcam functioned with contactless operation and only required the users to stand on the mat for the image to be displayed on the screen. The UV webcam was designed to be plugged into any monitor or display screen with a high-definition multimedia interface connection point and display the image using a plug-and-play approach without requiring any software or an internet connection.

#### Observational Testing

To check whether the UV webcam was connecting and recording use data from the pressure sensor mat correctly, observational testing was performed in Brisbane, Australia (approximate latitude 27°S, 153°E). A total of 2 volunteers (90 kg and 60 kg) stood on the pressure sensor mat and used the UV camera 10 times, and the time-stamped data collected by the device were then compared with observational data.

#### Safety Testing

The temperature of the UV webcam device after 2 and 4 hours of continuous operation was recorded using an infrared handheld thermometer (ThermaTwin TN410LCE Infrared Thermometer). The UV radiation emitted by the UV light source was measured using a UV intensity meter (Solar Light Co, model PMA2100) fitted with a digital sensor (Solar Light Co, model PMA2101). The detector head of the sensor was positioned at a distance where a person’s face would be placed during use.

#### Field-Testing

A field study was conducted from November 21 to 27, 2020, in spring in Australia. The UV webcam was deployed to Surfers Paradise beach in Queensland, Australia (approximate latitude 28.0°S, 153.4°E). The UV webcam was placed near the beach entry on the esplanade in a high-traffic area accessed by the public. The UV webcam was deployed at the start of the day until the end of daylight hours, and free SPF 50+ chemical sunscreen was available next to the UV webcam through a touchless automatic dispenser system (Danger Sun Overhead). End users could provide optional feedback if desired using the contact email and phone number provided next to the UV webcam. The deployment of the UV webcam was to assess the functionality and not human subjects’ research; therefore, we obtained an institutional ethics review board exemption from the Human Research Ethics Committee of the Queensland University of Technology for this phase of the study.

Weather measurements were collected during the field study. Temperature data were recorded in degrees Celsius for the daily minimum and maximum as well as for observations at 9 AM and 3 PM each day. The temperature data were captured by the Bureau of Meteorology weather station (no.: 040764; Gold Coast Seaway, latitude 28°S, 153°E). The UV radiation data were captured by the Australian Radiation Protection and Nuclear Safety Agency detector (Gold Coast, latitude 28°S, 153°E), with the standard erythemal dose (SED) calculated with daily summaries and hourly observations recorded at 10 AM and noon.

## Results

### Phase 1: Laboratory Testing of UV Cameras

**Figure 1:**
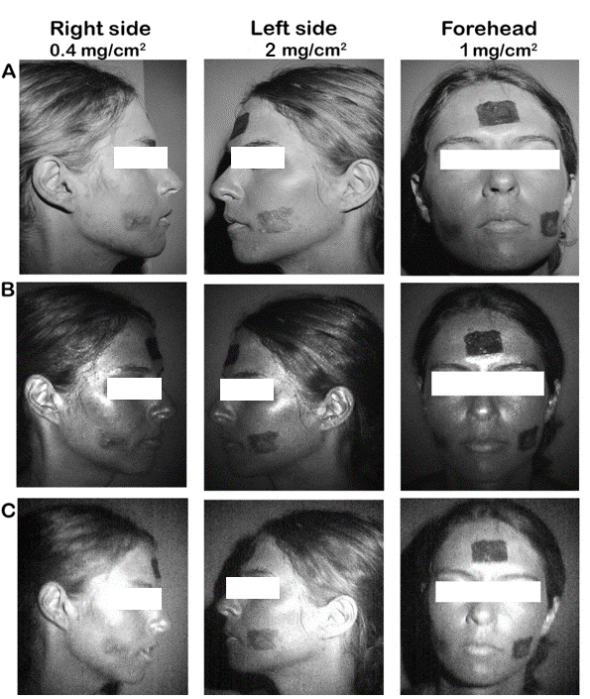
Comparison of UV photography devices. (A) A digital single-lens reflex (DSLR) UV camera, (B) Nurugo sun protection factor (SPF) camera, and (C) Sunscreenr camera were used to capture images of a SPF 50+ lotion applied to a 4 cm×2.5 cm area at a set concentration on the right cheek (0.4 mg/cm^2^), forehead (2 mg/cm^2^), and left cheek (1 mg/cm^2^).

A total of 10 participants enrolled and completed the laboratory testing phase. The participants were mostly female (8/10, 80%), and 70% (7/10) of the participants had very fair or fair skin (Table S2 in Multimedia Appendix 1). All 3 UV cameras captured well-defined areas when the SPF lotions were applied at concentrations of 2 and 1 mg/cm^2^. Figure 1 shows the areas where SPF lotions were applied to the participants’ faces, with the dark areas indicating SPF lotions are present.

The quality of the image captured by the DSLR UV camera was the highest of the 3 UV cameras purchased, with an image size of 6000×4000 pixels and a resolution of 300 dpi (dots per inch). The image size captured by the Sunscreenr camera was 1716×1290 pixels at 72 dpi and by the Nurugo SPF camera was 480×640 pixels at 72 dpi. Both the Nurugo SPF and Sunscreenr cameras collected images that had sufficient image quality for an observer to view the images on the small screen of a smartphone.

With the 10 volunteers, the sensitivity of the UV camera devices was also tested using 3 SPF-containing lotions applied at concentrations of 2, 1, and 0.4 mg/cm^2^ (Figure S2 in Multimedia Appendix 1). There was perfect agreement (100%) across the UV camera devices when the concentration of the SPF product was high (2 and 1 mg/cm^2^; Table 1). The lower 0.4 mg/cm^2^ application thickness had less coverage, but there was still strong agreement among the UV camera devices (Table 1).

**Table 1:** The percentage of coverage at each treatment site determined by 3 UV camera devices in 10 volunteers.

Sun protection product			DSLR^a^ UV camera (n=10); % (SE)		Sunscreenr UV camera (n=10); % (SE)		Nurugo SPF^b^ camera (n=10); % (SE)
**Sunscreen SPF 50+ (mg/cm^2^)**							
	2	100 (0)		100 (0)		100 (0)	
	1	100 (0)		100 (0)		100 (0)	
	0.4	72 (3.1)		72 (3.1)		73 (3.4)	
**Moisturizer SPF 50+ (mg/cm^2^)**							
	2	100 (0)		100 (0)		100 (0)	
	1	100 (0)		100 (0)		100 (0)	
	0.4	69 (0.2)		68 (1.4)		69 (0.2)	
**Moisturizer SPF 15+ (mg/cm^2^)**							
	2	100 (0)		100 (0)		100 (0)	
	1	100 (0)		100 (0)		100 (0)	
	0.4	69 (0.3)		68 (1.3)		69 (0.3)	

^a^DSLR: digital single-lens reflex.

^b^SPF: sun protection factor.

### Phase 2: Testing UV Photography Using a Range of Sun Protection Products

#### Part 2a: Observational Study

A total of 39 participants enrolled and completed the morning photo session, and 2 participants did not return for their afternoon photos. Furthermore, 2 participants wore products that contained only physical active ingredients and were excluded from further analysis (Figure S3 in Multimedia Appendix 1). Overall, 35 participants were included in the analysis.

The participants were mostly females (34/35, 97%), and 63% (22/35) of the participants had very fair or fair skin type (Table S2 in Multimedia Appendix 1). Just over half of the participants had applied 1 SPF-containing product (20/35, 57%), 34% (12/35) of the participants had applied 2 products, and 9% (3/35) of the participants had 3 or more products applied to their faces. The most used type of product was facial moisturizer (22/55, 40%), followed by liquid foundation (17/55, 31%), sunscreen (11/55, 20%), lip balm/lipstick (3/55, 5%), and powder foundation (2/55, 4%). Of the 55 facial products used by the participants, 25% (14/55) were SPF 50+, 53% (29/55) were SPF 15+, 4% (2/55) were below SPF 15, and 18% (10/55) had no SPF rating. A total of 66% (23/35) participants used one or more products that were tinted and contained chemical UV filters as well as varying quantities of titanium dioxide or zinc oxides.

Participants reported high satisfaction with the UV photography images, with 83% (29/35) of participants likely to use UV photography in the future to help guide the application of SPF products, whereas 80% (28/35) of participants would share their UV image with friends or family (Table S2 in Multimedia Appendix 1).

Participants’ images captured in the morning showed good coverage of sun protection products on their nose (32/35, 91%), and 80% (28/35) of participants had their cheeks covered, and 71% (25/35) of the participants had their forehead protected. By the afternoon, the coverage of sun protection products had decreased, with only 74% (26/35) of the participants still having good coverage on their nose, 63% (22/35) having their cheeks protected, and 51% (18/35) having their forehead covered. On average, the morning and afternoon photos were taken 4 hours and 37 minutes apart. Commonly missed areas included the medial canthal area (across the eyes), which was missed by 37% (13/35) of the participants in the morning, and by the afternoon, 69% (24/35) of the participants had no sun protection in this area (Figure S4 in Multimedia Appendix 1).

#### Part 2b: Testing Tinted SPF Lotions

**Figure 2:**
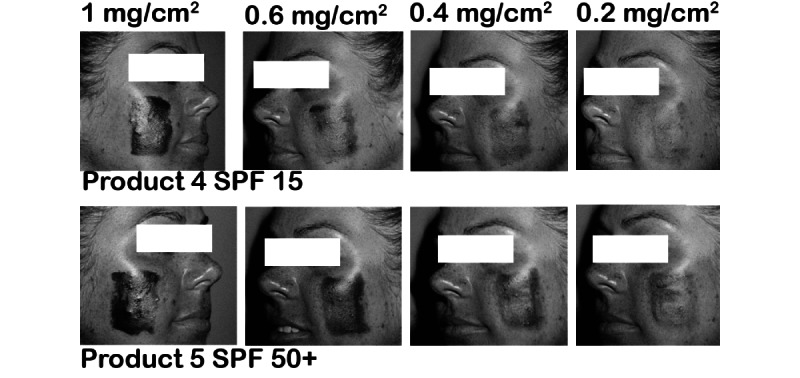
Tinted cosmetics and sun protection coverage using UV photography. The top panel shows product 4 applied to the cheek at concentrations of 1, 0.6, 0.4, and 0.2 mg/cm^2^, and the bottom panel shows product 5 applied at the same concentrations. SPF: sun protection factor.

The level of coverage varied greatly between the 5 tinted products, regardless of SPF rating (Figure S5 in Multimedia Appendix 1). Overall, 3 out of the 5 tinted SPF products were barely visible or not visible using UV photography. A review of the product ingredient list revealed that all the 5 products tested listed chemical UV filters as well as varying quantities of titanium dioxide or zinc oxides. UV photography was shown not to be suitable for 3 of the tinted products, as the physical blockers titanium dioxide or zinc oxides may have affected the ability to capture UV images. However, 2 tinted sun protection products were visible using UV photography even at low concentrations. The level of coverage was still high for the SPF 50+ product, yielding a dark area at the treatment site even when applied at 0.2 mg/cm^2^ (Figure 2). Although the SPF 15 product had adequate coverage at 1 mg/cm^2^, the absorption of UV was reduced at the lower 0.6, 0.4, and 0.2 mg/cm^2^ sites (Figure 2).

### Phase 3: Development, Safety, and Field-Testing of a UV Webcam

#### Development

**Figure 3:**
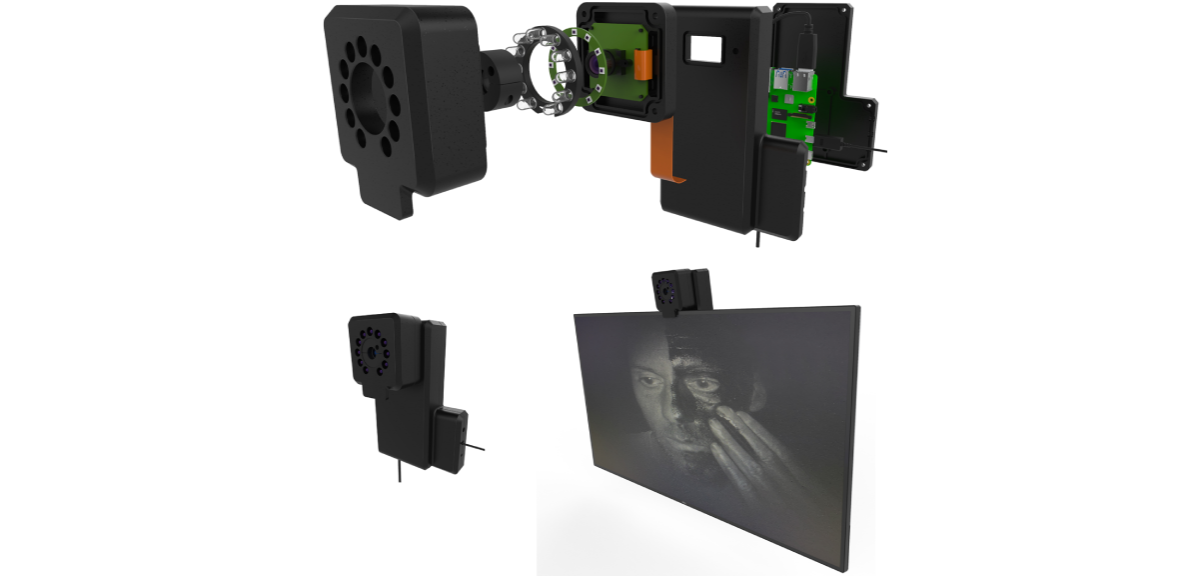
UV webcam device. The top panel shows the electrical components, which are housed within a plastic box (bottom left panel) and mounted on a monitor connected via a high-definition multimedia interface cable to display UV images (bottom right panel). The darker areas on the face show where sunscreen has been applied.

The prototype UV webcam was developed to provide personalized feedback about where improvements could be made for sunscreen application (Figure 3).

#### Observational Testing

The UV webcam was able to track use through a pressure-sensitive mat. Testing demonstrated perfect agreement with observed use and device-recorded use, with a κ value of 1.0, and the 95% CI range was 1.0-1.0 (Table 2).

**Table 2:** Agreement between observed use and UV webcam–recorded use (N=10).

Weight of person using UV webcam (kg)	UV webcam device–reported use, n (%)	
	Yes	No
90 (observed use=10)	10 (100)	0 (0)
60 (observed use=10)	10 (100)	0 (0)

#### Safety Testing

The prototype unit emits only UV-A irradiation and requires over 7 hours of continuous exposure to equal 15 minutes of midday sun light in Brisbane, Australia. During observational testing and field-testing, no eye or skin irritation was reported or observed using the UV webcam. The temperature of the UV webcam device after 2 and 4 hours of continuous operation was 37.1°C and 37.7°C, respectively.

#### Field-Testing

The UV webcam recorded data each day and was used 233 times during deployment at the beach location (Figure S6 in Multimedia Appendix 1). The UV radiation exposure level was consistently high, requiring sun protection each day during the field-testing, with daily SEDs ranging from 56 SEDs to 70 SEDs (Table S3 and Figure S7 in Multimedia Appendix 1). The UV index level was above 3 for over 5 hours each day during the field-testing, and no daytime rainfall was recorded (Table S3 in Multimedia Appendix 1). The average daily maximum temperature was 27.6°C (range 18.9-30) during field-testing (Table S3 in Multimedia Appendix 1).

No complaints, adverse events, or concerns were logged from users during the 7 days when the UV webcam was deployed. The UV webcam had sufficient power to function via a USB connection, and the display monitor required a 240-volt power supply.

## Discussion

### Principal Findings

Our study investigated the use of UV photography as a sun safety educational approach. A total of 3 UV camera systems were tested, and all devices identified the application of a range of SPF 15 to 50+ chemical sunscreens, moisturizers, and cosmetics. The sensitivity of the UV camera devices was shown to be adequate, with SPF-containing products applied at concentrations of 2 and 1 mg/cm^2^ clearly visible and SPF-containing products applied at a concentration of 0.4 mg/cm^2^ having lower levels of coverage. This study showed that participants were engaged with the personalized feedback approach of UV photography and that a UV webcam connected to a large monitor was used by beachgoers. However, we found that 3 out of the 5 tinted SPF products were not visible using UV photography because of the varying quantities of titanium dioxide and zinc oxides, which limit the sensitivity of UV photography. We recommend the use of UV photography for translucent sun protection lotions, including chemical sunscreens and moisturizers, and found that UV photography is less reliable for tinted products.

Sunscreens and SPF-containing moisturizers or cosmetics are commonly used for sun protection. In Australia, a cohort study of over 40,000 respondents reported that 40% regularly used sunscreen or cosmetics with SPF on their faces [16]. Young adults in a holiday beach setting reported high rates of daily sunscreen use (166/188, 88.3%), and most participants who reported being sunburnt also reported applying sunscreen [17]. Further data also suggest that both adults and children apply far less sunscreen than recommended, resulting in less protection [18,19]. The effectiveness of SPF-containing lotions depends on the application thickness, covering all sun-exposed skin, and regular reapplication [20]. In phase 2, we found that the SPF products had less coverage on the face by the afternoon compared with the morning photo session several hours earlier. Here, we showed that UV photography is a practical, well-liked method to visualize the need for sunscreen reapplication, with most participants indicating that they would use the technology again in the future. An estimated 7220 melanoma cases are attributed to sun exposure in Australia each year, and the effective use of sunscreen could reduce this burden, with health interventions using UV photography offering substantial opportunity for improvement [21].

Strategies to improve sunscreen application are important, as the belief that the whole face or body is protected following an application may increase UV exposure [22]. Previous research using UV photography has found that individuals do not apply sunscreen uniformly across the whole body [18]. A total of 52 participants were asked to apply sunscreen on their whole body, and researchers found that sunscreen application on the front side of the body was better than the back, and females covered their skin better than males [18]. UV photography may assist sunscreen application by providing personalized feedback on missed areas as well as revealing when reapplication is required.

We developed a UV webcam device and deployed it in a high UV environment and found that it was used by beachgoers during the weeklong field test. We chose a beach setting to deploy the UV webcam device because of the high rates of sunburn in these environments. In Queensland, 45% of children reported being sunburnt in the previous 12 months, and 69% of these sunburns happened during a water-based activity [8]. Future research could explore whether beachgoers improve their sunscreen application following personalized feedback from the UV webcam and explore the effect this technology may have on reducing sunburn. Previous research has shown that the benefits of ecological momentary health interventions, which influence behaviors within an environmental context, can improve willingness to change behavior [23].

In addition to visualizing sunscreen coverage, UV photographic imaging has also been shown to be a beneficial tool for assessing skin damage and promoting behavioral change by highlighting the negative effects of the sun on an individual’s appearance in sun bed users [24] and young adults [25,26]. To further engage the public, recent strategies by Cancer Council Western Australia have included UV camera imagery to raise awareness of sun damage [27]. UV photography is a valuable public health promotional tool, and it is also a convenient method for use in a research setting. Other methodologies to assess sunscreen application include tape stripping, swabbing of body sites, and laboratory processing of samples using fluorescence spectroscopy, which can be laborious and time-consuming.

A limitation of UV photography imaging is the use of SPF lotions containing physical blockers such as titanium dioxide or zinc oxide, which are not detected. Several tinted foundations use titanium dioxide as an ingredient but have additional chemical filters to reach the stated SPF rating; however, these combination cosmetic products did not perform well in testing, with 3 out of 5 products not being detected by UV photography. Limitations of this study include selection bias, as participants in the observational study were recruited using a convenience sample, and we did not use a random sampling method. Phase 1 and 2 participants were mainly female and therefore may not represent the general population. In phase 2, under the study conditions, participants might have been more cautious than real life and applied SPF lotions more carefully. In phase 3, we did not capture self-reported sunburns or behavioral changes from participants.

### Conclusions

Reducing the number of Australians sunburnt each year forms a crucial part of sun safety initiatives, and improving the messaging on the quantity of sunscreen to apply to achieve sufficient coverage as well as commonly missed areas is essential. In this study, we tested a variety of UV cameras and found that UV photography could identify the application of SPF-containing chemical filter sunscreens and moisturizers as well as determine unprotected areas. We found that the participants were engaged with personalized UV photography feedback.

## Appendix

Multimedia Appendix 1 Additional research and methods information.

## Abbreviations

BB: beauty balm
dpi: dots per inch
DSLR: digital single-lens reflex
SED: standard erythemal dose
SPF: sun protection factor
