# Supplementary material for: Assessing Sunscreen Protection Using UV Photography: Descriptive Study
Source: JMIR Dermatol. 2021 May 26;4(1):e24653. doi: 10.2196/24653 (PMC10501517; doi:10.2196/24653)
Supplement: Multimedia Appendix 1 [file derma_v4i1e24653_app1.docx]

**Supplementary Materials**


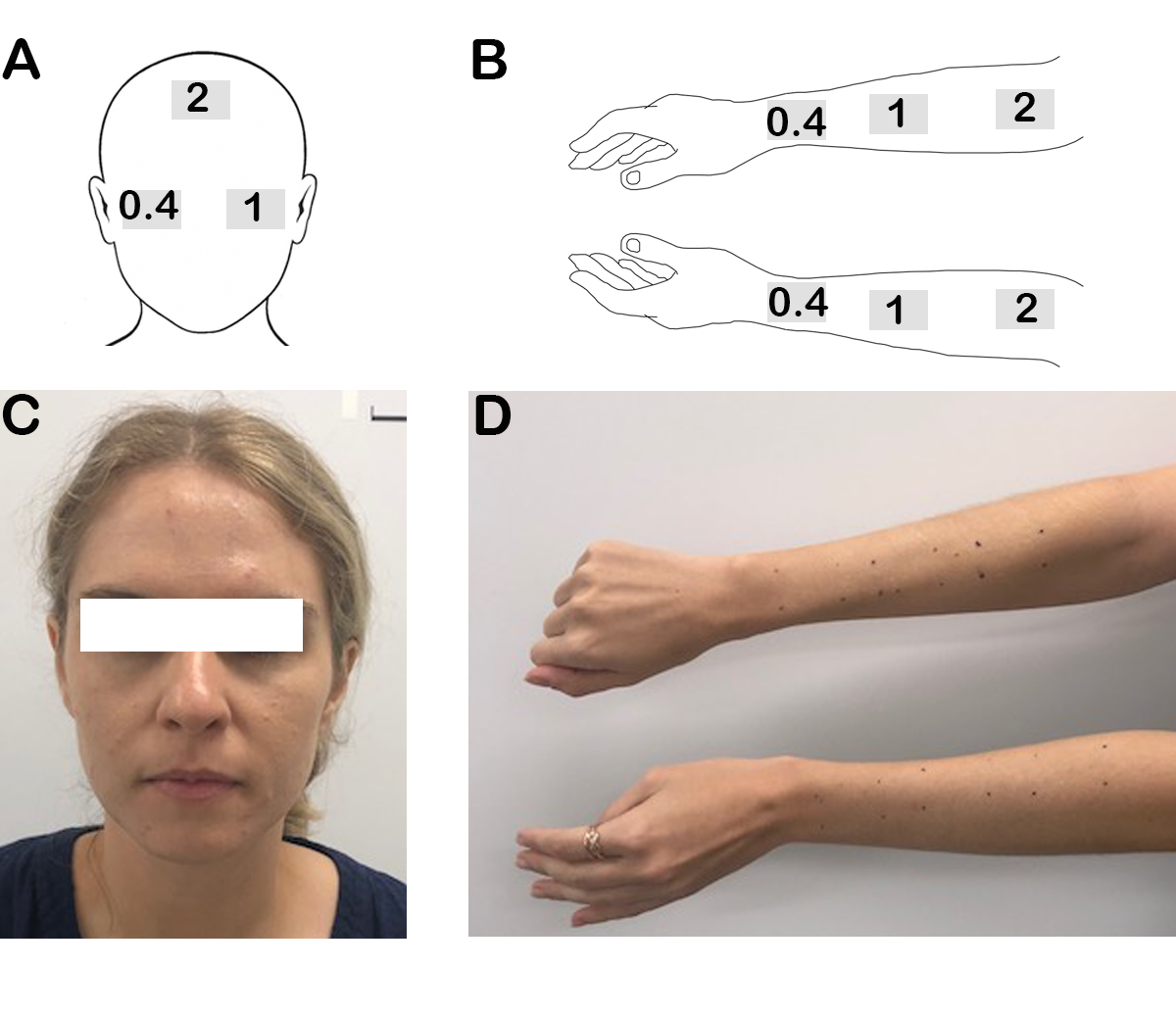


**Figure S1. Application of SPF-containing products to body sites.** SPF containing products were applied by the researcher at set concentrations to A) face and B) forearms, the researcher equally disturbed the SPF containing product across a 4cm x 2.5cm region at the set concentrations of 2mg/cm^2^, 1mg/cm^2^, 0.4mg/cm^2^.


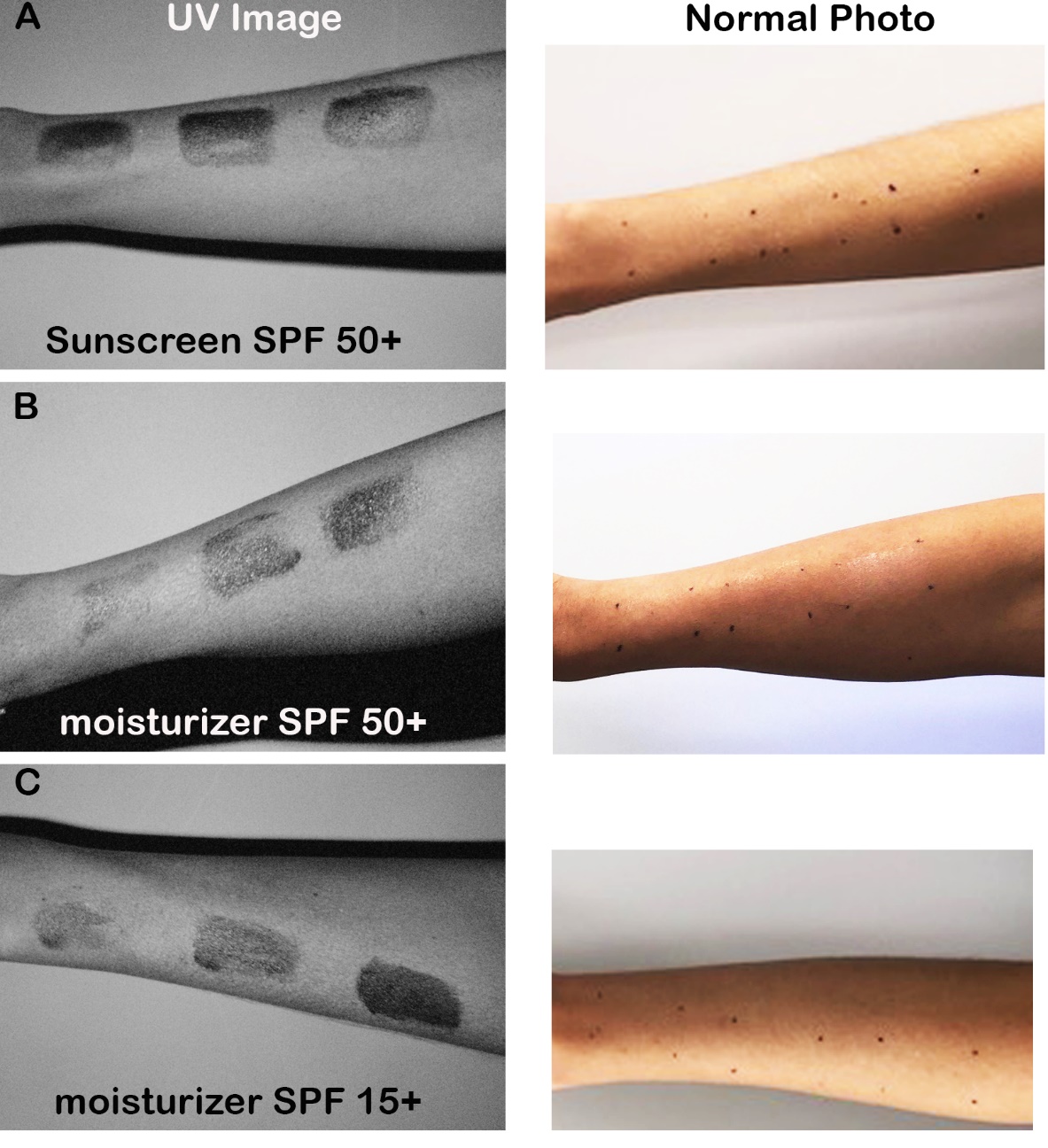


**Figure S2. Comparing SPF-containing products using UV photography.** Left panel shows UV camera captured image and right panel shows normal camera image; A) Sunscreen product with a SPF 50+ rating, B) Moisturizer SPF 50+ product and C) Moisturizer SPF 15+ product. Each product was applied at a set concentration starting at 0.4 mg/cm^2^ near the wrist, 1 mg/cm^2^ in the middle and 2 mg/cm^2^ near the elbow.

**UV photography morning**

**(n= 39)**

**Lost to Follow-up (n= 2)**

**UV photography afternoon**

**Participant shown photos and interview conducted**

**(n= 37)**

**Excluded (n= 2)***

**Image analysis**

**(n= 35)**

**Figure S3. Flow chart of observational study of indoor workers.**

*participants applied SPF products containing active ingredients zinc oxide or titanium dioxide, which are physical filters and are not detected using UV photography. These participants were excluded from further analysis.

**
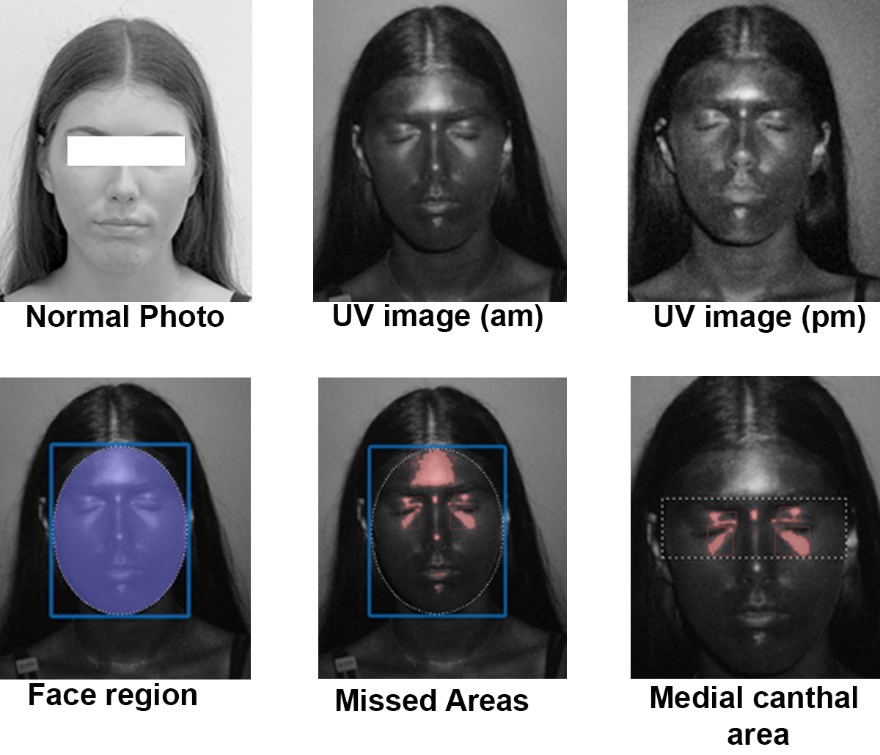
**

**Figure S4. Analysis of the UV images collected from indoor workers**. Top panel: left) Standard photograph of participants’ face, centre) UV photograph of participants’ face in the morning and right panel UV image collected in the afternoon. The bottom panel illustrates the image analysis process, left) the region of interest detected and highlighted in blue, centre) areas on the face not adequately covered highlighted in red, right) the medial canthal area with low coverage shown in red.

**
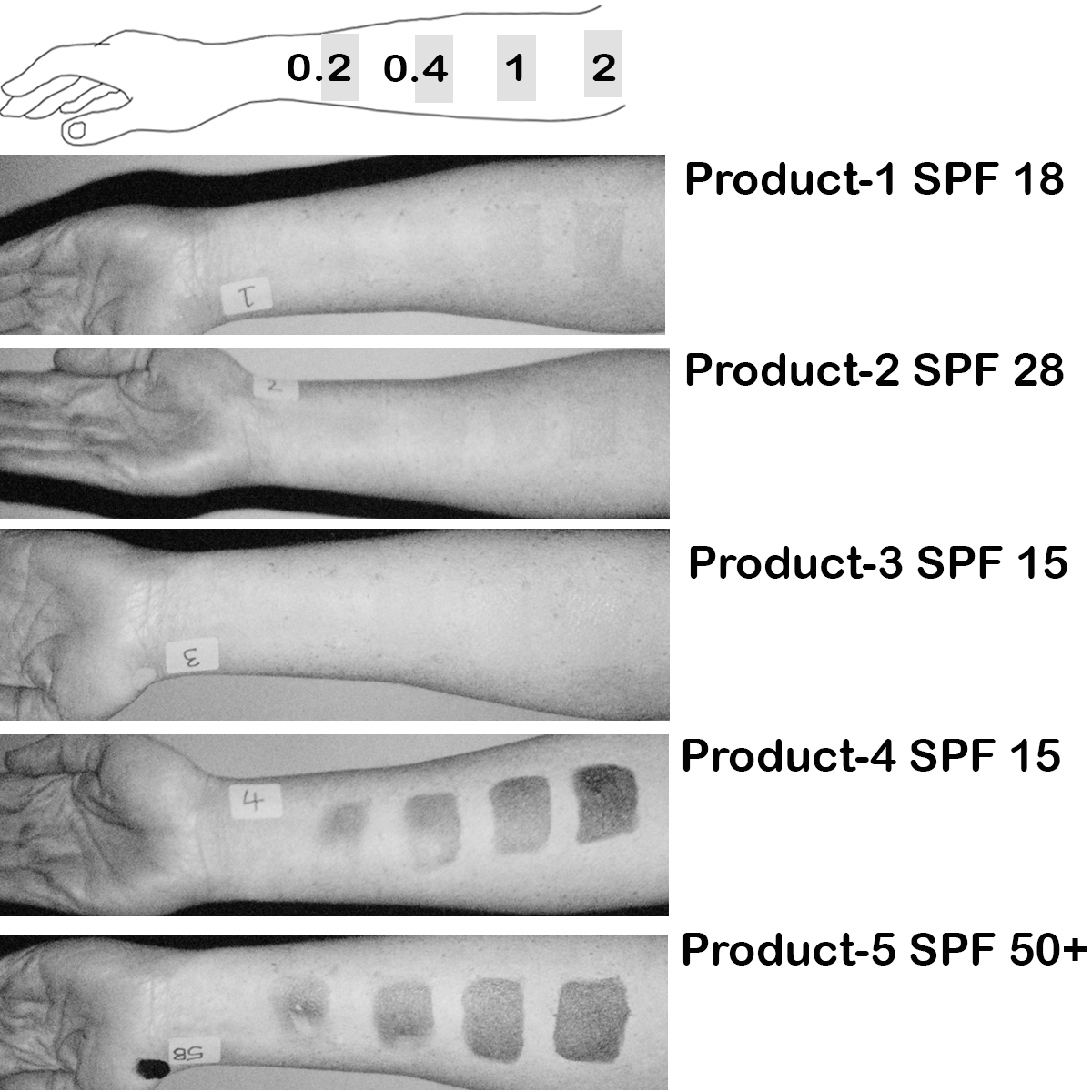
**

**Figure S5. Tinted sun protection products applied to the forearm at a series of concentrations.** Five tinted products were applied at 2 mg/cm^2^, 1 mg/cm^2^, 0.4 mg/cm^2^ and 0.2 mg/cm^2^ to a 4cm x 2.5cm area of skin and images were collected using the DSLR UV camera.

**
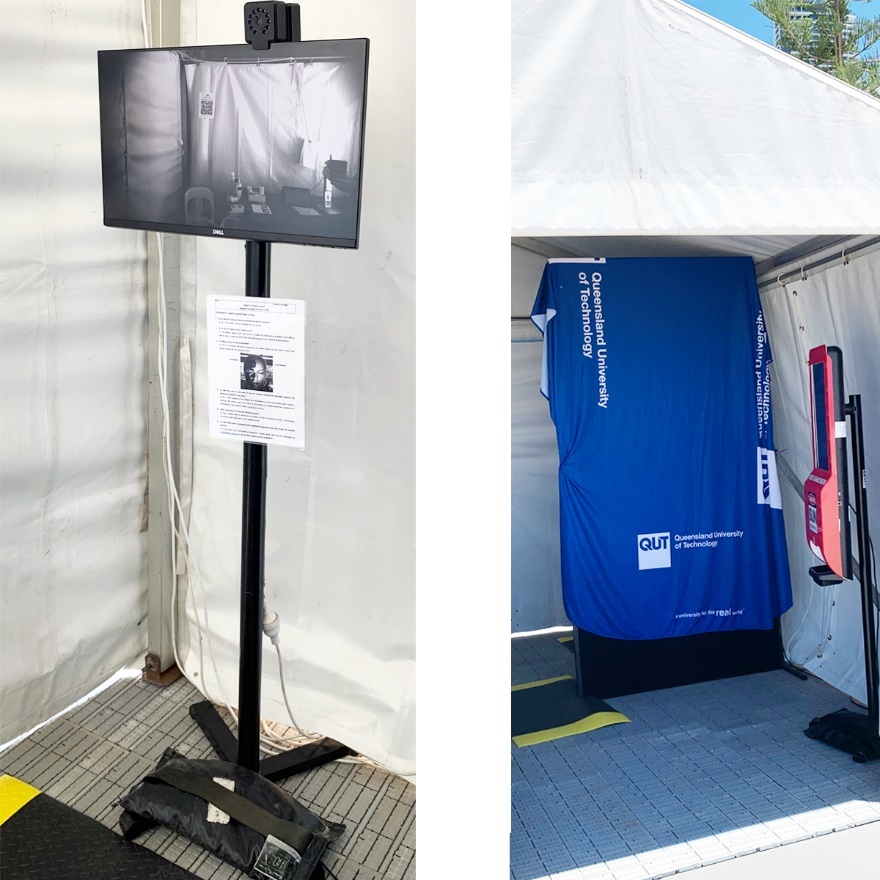
**

**Figure S6. Field testing of the UV webcam device.** The UV webcam was setup in a photo booth marquee next to an automatic sunscreen dispenser on the esplanade of Surfers Paradise beach.

**
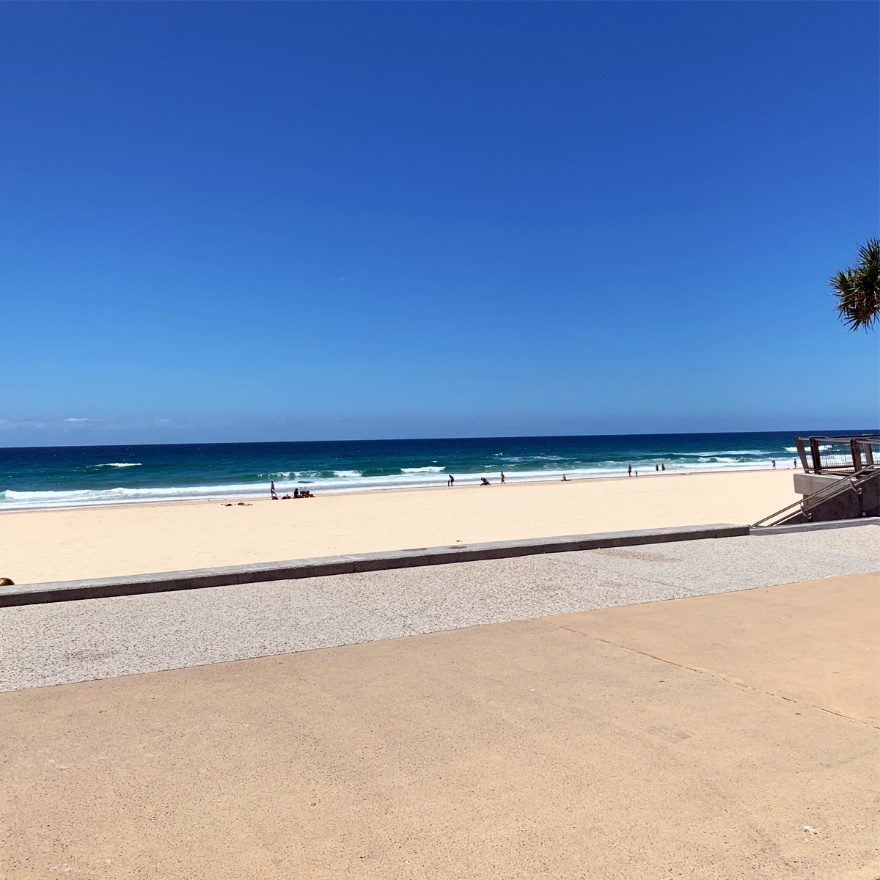
**

**Figure S7. The beach location where fielding testing was undertaken.** The photo booth marquee was setup on the esplanade across at a pedestrian entry point to the Surfers Paradise beach, Australia.

**Table S1. Indoor workers interview data on UV photography**

|  | |  | **Total**  **n=35** | |
| --- | --- | --- | --- | --- |
|  | |  | n | (%) |
| Would you like to use a UV camera in the future to help test cosmetic products at the point of purchase? | | |  |  |
|  | Yes | | 28 | (80.0) |
|  | No | | 6 | (17.1) |
|  | Unsure | | 1 | (2.9) |
| Would you like to use a UV camera in the future to help guide sunscreen application? | | |  |  |
|  | Yes | | 29 | (82.9) |
|  | No | | 6 | (17.1) |
|  | Unsure | | - |  |
| Did it make you feel uncomfortable looking at the photos of yourself? | | |  |  |
|  | Yes | | 10 | (28.6) |
|  | No | | 20 | (57.1) |
|  | Unsure | | 5 | (14.3) |
| Would you show the photograph to others? | | |  |  |
|  | Yes | | 28 | (80.0) |
|  | No | | 6 | (17.1) |
|  | Unsure | | 1 | (2.9) |
| Were you surprised by the coverage? | | |  |  |
|  | Yes | | 24 | (68.6) |
|  | No | | 10 | (28.6) |
|  | Unsure | | 1 | (2.9) |
| Can you see a difference in coverage between the morning and afternoon photos? | | |  |  |
|  | Yes | | 28 | (80.0) |
|  | No | | 7 | (20.0) |
|  | Unsure | | - |  |
| Are you confident in your cosmetic products tested? | | |  |  |
|  | Yes | | 21 | (60.0) |
|  | No | | 7 | (20.0) |
|  | Unsure | | 7 | (20.0) |

**Table S2. Participant characteristics**

|  | |  | **Phase 1**  **Testing UV cameras n=10** | | **Phase 2**  **Indoor workers**  **n=35** | |
| --- | --- | --- | --- | --- | --- | --- |
|  | |  | n | % | n | (%) |
| **Age mean (range)** | | | 40.2 (20-57) | | 41.3 (25-62) | |
| **Gender** | | |  |  |  |  |
|  | Female | | 8 | (80.0) | 34 | (97.1) |
|  | Male | | 2 | (20.0) | 1 | (2.9) |
| **Skin colour** | | |  |  |  |  |
|  | Very Fair | | 3 | (30.0) | 6 | (17.1) |
|  | Fair | | 4 | (40.0) | 16 | (45.7) |
|  | Medium | | 3 | (30.0) | 10 | (28.6) |
|  | Olive/Dark | | - |  | 3 | (8.6) |
| **Natural hair colour at 18 years** | | |  |  |  |  |
|  | Red/auburn/blonde | | - |  | 4 | (11.4) |
|  | Light brown/ dark brown | | 9 | (90.0) | 25 | (71.4) |
|  | Black | | 1 | (10.0) | 6 | (17.1) |
| **How much would your skin burn in strong summer sun for 30 minutes in the middle of the day without sun protection?** | | |  |  |  |  |
|  | My skin would not burn at all | |  |  |  |  |
|  | My skin would burn lightly | | 3 | (30.0) | 13 | (37.1) |
|  | My skin would burn moderately | | 3 | (30.0) | 14 | (40.0) |
|  | My skin would burn severely | | 4 | (40.0) | 8 | (22.9) |
| **Previous skin cancer** | | |  |  |  |  |
|  | Yes | | 2 | (20.0) | 6 | (17.1) |
|  | No | | 8 | (80.0) | 29 | (82.9) |
|  | Unsure/don’t know | |  |  |  |  |

**Table S3. Weather conditions during the field testing.**

| Date | Start of Day when UVI >3 | End of Day  when UVI  <3 | Temperature | | | | UV  Daily^a^ Dose | UV 10-11am^b^ Dose | UV 12-1pm^c^ Dose | Rain (mm) |
| --- | --- | --- | --- | --- | --- | --- | --- | --- | --- | --- |
|  |  |  | Min | Max | 9AM | 3PM |  |  |  |  |
|  |  |  | °C | °C | °C | °C | SEDs | SEDs | SEDs |  |
| 21-Nov-20 | 7:30 | 15:30 | 19.3 | 27.3 | 26.2 | 25.7 | 70 | 11 | 11 | 0 |
| 22-Nov-20 | 7:30 | 15:30 | 18.9 | 28.9 | 26.9 | 27.5 | 70 | 11 | 11 | 0 |
| 23-Nov-20 | 8:00 | 15:30 | 20.0 | 30.0 | 26.0 | 25.3 | 68 | 10 | 10 | 0 |
| 24-Nov-20 | 8:00 | 14:00 | 22.0 | 26.0 | 24.3 | 23.4 | 58 | 10 | 9 | 0 |
| 25-Nov-20 | 9:00 | 14:30 | 20.2 | 26.3 | 23.6 | 23.9 | 56 | 5 | 4 | 1.4 |
| 26-Nov-20 | 7:30 | 15:00 | 18.9 | 27.3 | 26.0 | 25.2 | 64 | 11 | 11 | 0 |
| 27-Nov-20 | 7:30 | 15:00 | 19.4 | 27.4 | 25.7 | 25.6 | 60 | 10 | 8 | 0 |

UVI=Ultraviolet Index

SEDs=Standard Erythemal Dose

**^a^**= Daily dose calculated from 6:00-16:00

**^b^**= Morning dose calculated from 10:00-11:00

**^c^**= Midday dose calculated from 12:00-13:00
